# Synchronized Audio-Visual Transients Drive Efficient Visual Search for Motion-in-Depth

**DOI:** 10.1371/journal.pone.0037190

**Published:** 2012-05-17

**Authors:** Marina Zannoli, John Cass, Pascal Mamassian, David Alais

**Affiliations:** 1 Université Paris Descartes, Sorbonne Paris Cité, Paris, France; 2 Laboratoire Psychologie de la Perception, CNRS UMR 8158, Paris, France; 3 School of Psychology, University of Western Sydney, Sydney, New South Wales, Australia; 4 School of Psychology, University of Sydney, Sydney, New South Wales, Australia; CSIC-Univ Miguel Hernandez, Spain

## Abstract

In natural audio-visual environments, a change in depth is usually correlated with a change in loudness. In the present study, we investigated whether correlating changes in disparity and loudness would provide a functional advantage in binding disparity and sound amplitude in a visual search paradigm. To test this hypothesis, we used a method similar to that used by van der Burg et al. to show that non-spatial transient (square-wave) modulations of loudness can drastically improve spatial visual search for a correlated luminance modulation. We used dynamic random-dot stereogram displays to produce pure disparity modulations. Target and distractors were small disparity-defined squares (either 6 or 10 in total). Each square moved back and forth in depth in front of the background plane at different phases. The target’s depth modulation was synchronized with an amplitude-modulated auditory tone. Visual and auditory modulations were always congruent (both sine-wave or square-wave). In a speeded search task, five observers were asked to identify the target as quickly as possible. Results show a significant improvement in visual search times in the square-wave condition compared to the sine condition, suggesting that transient auditory information can efficiently drive visual search in the disparity domain. In a second experiment, participants performed the same task in the absence of sound and showed a clear set-size effect in both modulation conditions. In a third experiment, we correlated the sound with a distractor instead of the target. This produced longer search times, indicating that the correlation is not easily ignored.

## Introduction

For the last fifty years [1], visual search paradigms have proven to be a useful tool to study feature integration [2] and allocation of attention [3]. A majority of studies using this paradigm have focused on the processing of basic feature dimensions such as luminance, color, orientation or motion, and have shown that searching for a target which is distinguished from the surrounding distractors by having, for example, a different orientation (or color, or luminance, etc) produces fast, efficient searches. Most visual search studies employ 2D arrays and relatively few have examined visual search in the 3D domain. Of these, an early study by Nakayama & Silverman [4] showed that distinguishing targets and distractors by their horizontal binocular disparity (stereopsis) was sufficient to support efficient visual search. Later, Harris, McKee & Watamaniuk [5] found that when binocular disparity was defined by spatiotemporal correlations (i.e., perceptual stereomotion), search performance became far less efficient. That is, stereomotion did not support pop-out. This is an intriguing result because even though static stereopsis and stereomotion are each capable of supporting vivid and clearly discriminable perceptual structure, stereomotion seems to require serial search.

In the present study, we will investigate whether search efficiency for stimuli defined by stereomotion can be improved by a non-spatial auditory cue correlated with the visual target. The ability of auditory signals to improve visual processing is now well known. Several studies have shown that the presentation of a simultaneous sound can improve visual performance for detection [6] can increase the saliency of visual events [7] and can drive visual attention [8]. More specifically, using the visual search paradigm, van der Burg and colleagues recently conducted a series of studies on the so-called “pip and pop” effect and demonstrated that a synchronized, but spatially nonspecific, sound can drastically improve search efficiency as long as the visual signal is temporally abrupt [9]–[11]. In the so-called “pip and pop” effect, search times are drastically decreased for visual objects that are synchronized with an auditory beep even though the sound contains no spatial or identity information concerning the visual target. According to van der Burg and colleagues the auditory “pip” and the visual target are integrated, creating a salient audiovisual object that draws exogenous attention. To test the effect of an auditory cue on visual search for stereomotion stimuli, we used a method similar to the one introduced by van der Burg et al. [10].

The study by van der Burg et al. [10] demonstrated that non-spatial modulations of loudness can drastically improve spatial visual search for a correlated *luminance* modulation but that it requires *transient* visual events (square modulations instead of sine) to elicit efficient search. To enable a comparison with the findings of Van der Burg, et al. [10] in the luminance domain, we decided to use similar modulation conditions. Our participants were presented with a dynamic random dot stereogram [12] in which 6 or 10 disparity-defined squares arranged on a ring moved back and forth in depth in front of the background plane. Critically, elements in these displays are invisible when viewed monocularly, and require binocular integration across multiple frames. All the elements followed the same spatio-temporal modulation frequency but with different phases. An amplitude-modulating auditory beep was synchronized with the on of the elements’ depth modulation. Following the lead of van der Burg, et al. [9], [10] we employed a compound search task in which participants performed a discrimination task on a luminance-defined target. The discrimination task is unrelated to the stereomotion but does require participants to successfully find the sound synchronized visual element first.

Although our study uses similar experimental conditions to van der Burg et al. [2], different predictions can be made concerning the modulation conditions. In their study, search for luminance-defined targets was more efficient in the square-wave condition. In our experiment, because binocular matching processes are known to favor smooth over abrupt changes of disparity across space and time [13]–[15], we predict that the square-modulation condition will not suit stereo processing and will therefore lead to longer response times compared to the sine-modulation condition. In addition, we predict that the presence of the auditory cue will enhance search efficiency in the sine condition and produce smaller set-size effects.

## Materials and Methods

### Experiment 1

In the first experiment, we tested whether correlating changes in disparity and loudness would provide a functional advantage in binding disparity and sound amplitude in a visual search task. For this purpose, we used visual stimuli moving in depth together with an amplitude-modulating auditory sound with a static location. Participants had to perform a search and a spatial discrimination task on a small 2×2 pixel square defined by luminance. Participants were informed that this luminance target was adjacent to the visual element that was correlated with the accompanying sound changes.

#### Participants

Five observers (two naïve) with normal or corrected-to-normal vision were recruited in the laboratory building. All participants had experience in psychophysical observation and had normal stereo acuity and hearing. They all gave written informed consent before participating in the experiment.

#### Stimulus presentation

The stereograms were presented on a 21″ CRT monitor (Sony Multiscan G500, resolution 1024×768 pixels x 85 Hz, for four observers and ViewSonic 2100, resolution 1280×960×85 Hz for one observer) at a simulated distance of 57 cm. To avoid the issues raised by shutter or polarized glasses [16] we used a modified Wheatstone stereoscope. In this type of display, the images presented to the two eyes are completely independent and are presented in perfect synchrony. Each eye viewed one horizontal half of the CRT screen. A chin rest was used to stabilize the observer’s head and to control the viewing distance. The display was the only source of light and the stereoscope was calibrated geometrically to account for each participant’s interocular distance. The auditory stimuli were presented via a single loudspeaker, which was placed above the monitor.

#### Stimuli

Stereomotion can be extracted by computing interocular velocity differences and/or by tracking changes of disparity over time [12], [17]. In the first case, 2D motion is extracted for each monocular image and then compared between the two eyes’ images to compute speed and direction of motion. To avoid any 2D motion cues in the monocular components, we used dynamic random dot stereograms (DRDS). In DRDSs, the stereogram is rebuilt on each new video frame using a new pattern of random noise. Disparity is achieved by adding opposite disparity offsets to a small portion of the left and right images. Stereomotion is then obtained by smoothly changing the value of the disparity offsets from frame to frame. This way, stereomotion in our stimuli was entirely defined by changes of disparity over time. All Stimuli were generated using the Psychophysics Toolbox [18], [19].

**Figure 1:**
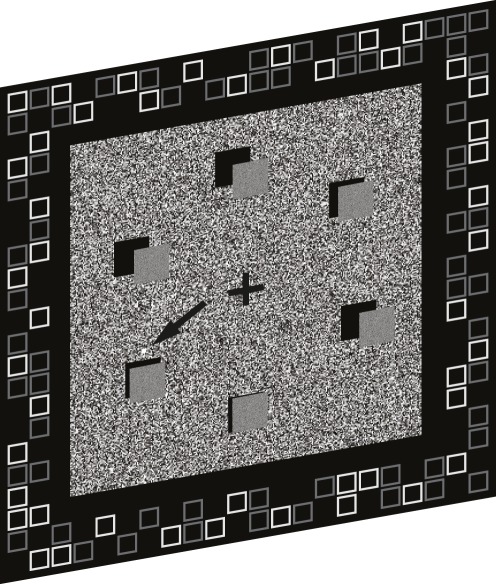
Perspective view of the stimulus used in all experiments. Visual elements were disparity-defined squares distributed evenly on a ring at 2.5 deg eccentricity and moved back and forth in depth from zero to +12 arcmin (crossed) disparity. The stimuli were surrounded by a vergence-stabilisation frame.

The background consisted of a 3.5×3.5 deg^2^ square of dynamic random noise (mean luminance 40 cd/m^2^; one-pixel resolution; refreshed every frame). Visual elements were 0.8×0.8 deg^2^ squares defined only by disparity and evenly presented on a virtual ring at 2.5 deg eccentricity. The number of elements was either 6 or 10. A small bright square (2×2 pixels, 80 cd/m^2^), too small to capture exogenous attention, was placed either above or below the sound synchronized disparity-defined square to enable a compound search task (see Procedure, below). The background was surrounded by a vergence-stabilization frame consisting of multiple luminance-defined squares (0.20×0.20 deg^2^; grey: 40 cd/m^2^ and white: 80 cd/m^2^) presented on a black background (5 cd/m^2^), with black nonius lines at the center (see Figure 1).

Visual elements moved in depth back and forth from 0 to +12 arcmin following a 0.7 Hz modulation. All elements moved at different phases. One of the squares’ depth modulation was synchronized with the sound amplitude modulation. To avoid overlapping temporal synchrony between the sound synchronized square and the other visual elements, we created an exclusion window of at least 60° around the sound synchronized square phase: for the other elements, phases were randomly assigned from the following values: ±60°, 80°, 100°, 120°, 140°, 160°, relative to the sound synchronized square’s phase.

The auditory stimulus was a 500 Hz sine-wave (44.1 kHz sample rate; mono) whose volume was modulated in amplitude (between 0 and 70 dB) at the same frequency as the visual motion-in-depth and synchronized with the square adjacent to the luminance target. The sound was presented over one loudspeaker placed on top of the CRT screen.

**Figure 2:**
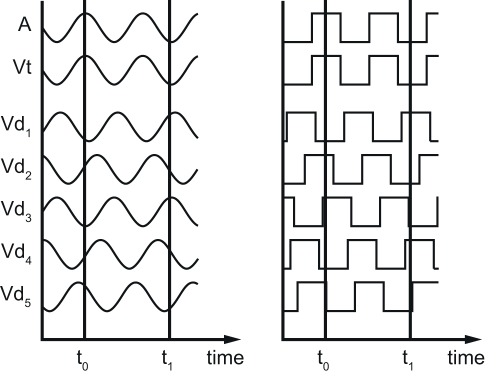
Audiovisual modulations. The depth modulation of the square adjacent to the luminance target is synchronized with an amplitude-modulated 500 Hz tone. Auditory and visual modulations are always congruent (both sine-wave or square-wave). A random phase is added to the AV modulation.

Both visual and auditory modulations were either sine-wave or square-wave and always congruent. A random phase was added to all modulations (see Figure 2). The auditory modulation was synchronized with the depth modulation of the disparity-defined square that was adjacent to the luminance target of the visual search.

#### Procedure

Participants were instructed to respond as fast as they could while maintaining good performance. Each trial started with a presentation of the nonius lines. When correctly fusing the nonius, participants pressed any key to start the stimulus presentation. In a speeded resnse task, the stimulus stayed on until participants had found the sound synchronized square and made the up/down judgment about the luminance target location and entered their answer on the keypad (which terminated the display). This up/down task (discriminating the position of the luminance target relative to the sound synchronized square) was orthogonal to the stereomotion search (locating the sound synchronized square), as it did not depend on the motion itself. However, as the luminance target was hardly visible while fixating centrally, the localization of the sound synchronized square was necessary first, before the up/down task could be done. This ensured that participants did perceive the disparity-defined squares.

Each combination of waveform condition (square vs. sine) and set size (6 vs 10) was repeated 80 times in total. The experiment was divided in ten sessions. Participants did not receive feedback regarding their accuracy, although they were aware that the amplitude modulation of the auditory signal was synchronized with the visual depth modulation of the adjacent square.

### Experiment 2

To test whether results obtained in Experiment 1 are due to the presence of a sound, we tested whether visual sine- and square-wave modulations would lead to different set-size effects in the absence of a congruent auditory modulation.

#### Method

For the second experiment, the five observers who participated in Experiment 1 (two of whom were naïve) were recruited for Experiment 2. Stimuli were presented using the same setup as in Experiment 1 and the stimuli were identical to the ones used in the first experiment. No auditory signal was presented. Visual elements moved in depth following the same modulation patterns as in Experiment 1. Instructions given to participants were identical to those in Experiment 1.

### Experiment 3

In the third experiment, we investigated whether observers were using a voluntary or automatic binding of audiovisual information. We tested this by measuring whether correlating the sound with a square that is not adjacent to the luminance target would lead to longer response times, using a cost-benefit paradigm similar to the one introduced by Posner [3]. In the cost-benefit paradigm, the subject has to perform a discrimination task on a target presented at different locations. Before the presentation of the target stimulus a cue is displayed briefly, indicating the location of the target for that trial. Posner demonstrated that presenting a valid cue (indicating the actual target location) led to shorter response times (i.e., a benefit), relative to a neutral cue (not indicative). On the contrary, presentation of an invalid cue (indicating a wrong location for the target) led to longer response times (i.e., a search cost).

We implemented a cost-benefit experiment in which the square-wave sound could be presented in synchrony with either the square adjacent to the luminance target or another square. 20% of trials were valid (i.e., the sound was synchronized with the adjacent square) and the remaining 80% were invalid trials (i.e., the sound was synchronized with one of the other squares). In invalid trials, if observers were automatically binding the auditory and visual information and going directly to the location where they were synchronized, they would be at a wrong location and would not find the small square there for the up/down discrimination task. They would then have to make a serial search around the depth-modulating visual squares until the one with the small square adjacent to it was found. For this reason, there would be a search cost for invalid if binding were automatic. Alternatively, if the binding of the sound and stereomotion signals were a voluntary strategy, it would be more strategic to ignore the audiovisual correlation (which would be beneficial in only 20% of trials) and begin each trial immediately with a serial search for the small square. If we observe a search cost in the invalid trials (i.e., a slowing of search times), it would show that audiovisual binding was automatic and difficult to ignore.

#### Method

The five observers who participated in the first two experiments were recruited for the third experiment. Stimuli were presented using the same setup as in the first two experiments. Visual stimuli consisted of nine elements (squares of 0.8×0.8 deg^2^) evenly distributed on a ring as in the first two experiments. Auditory stimuli were the same as in Experiment 1. Audiovisual modulations were similar to those in the first experiment (square vs. sine) except that the auditory signal was synchronized with the square adjacent to the luminance target modulation in only 20% of trials. In the remaining 80%, the sound was synchronized with one of the other eight squares. Instructions given to participants were identical as in the first two experiments.

## Results

### Experiment 1

Participants reported that they first localized the sound synchronized square and then saccaded to it to make the up/down judgment concerning the luminance target.

**Figure 3:**
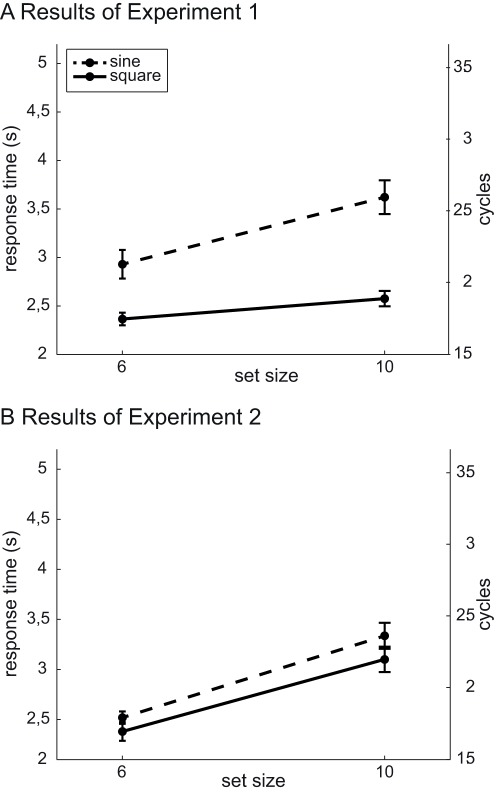
Results of Experiment 1 & 2. Mean response times pooled across five participants as a function of set size and waveform for Experiments 1 (a) & 2 (b). The y-axis on the right represents response times in number of cycles (at 0.7 Hz, 1 cycle lasts 1.4 s). The error bars reflect the overall standard errors of individuals’ mean response times. Dashed lines and solid lines code for sine-wave and square-wave modulations respectively.

**Figure 4:**
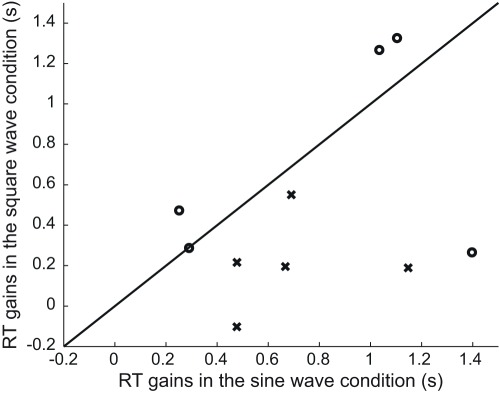
Individual results of Experiment 1 & 2. Response time (RT) gains ((RT(10) - RT(6)) in the square-wave condition as a function of the response time gains in the sine-wave condition. Along the black line, slopes are equal for both waveforms. When individual points are located in the lower part of the figure, response time gains are smaller in the square-wave condition. Crosses and dots represent individual results in Experiment 1 & 2 respectively.

Overall mean error rate was approximately 5% and error trials were discarded and no further analysis was conducted on those data. A cut-off was applied at two standard deviations from the mean response time for each participant (see Figure 3a and 4 and Table S1). A repeated-measures ANOVA was run on the response times with set size (6 vs. 10) and waveform (sine-wave vs. square-wave) as within-subject variables. The ANOVA revealed a significant main effects of set size (*F*(1, 3) = 25.9, *P*<0.01) and waveform (*F*(1, 3) = 15.7, *P*<0.05) and a significant interaction (set size x waveform) effect (*F*(3, 1) = 11.6, *P*<0.05).

#### Preliminary discussion

As shown in Figure 3a, the significant main effect of waveform arose because response times were faster in the square-wave condition overall. Interestingly, the set size effect was also reduced in the square-wave condition relative to the sine-wave condition. This indicates, contrary to our expectations, that visual search was faster and more efficient in the square wave condition.

In their 2010 study, van der Burg et al. [10] interleaved audiovisual trials with silent trials. This allowed them to interpret the set size effects observed in the audiovisual condition compared to the vision-only trials. During pilot experiments, our participants reported using two distinct conscious strategies depending on whether they were presented an audiovisual or a visual-only trial. Observers would wait for the sound to start to decide which strategy to use. In the presence of a visual-only trial, they would start serial searching for the luminance target while in the case of an audiovisual trial they would maintain central fixation and wait for the synchronized sound square to pop out. If observers were using distinct strategies depending on the condition, it seemed hazardous to compare data collected in the same experiment for these two sets of stimuli.

### Experiment 2

If the absence of a set-size effect observed in the square-wave condition in Experiment 1 were due to the auditory information, we expect no difference between the two modulation conditions in the absence of sound. If results from Experiment 2 are comparable to those obtained in Experiment 1, they might reflect a difference in task difficulty between the two modulation conditions. If the square-wave condition is very easy, we might observe a kind of “pop out” effect.

As in Experiment 1, overall mean error rate was approximately 5% and error trials were discarded. A cut-off was applied at two standard-deviations from the mean response time for each participant (see Figure 3b and 4 and Table S2). A repeated-measures ANOVA was run on the response times with set size (6 vs. 10) and waveform (sine-wave vs. square-wave) as within-subject variables. The ANOVA revealed only a significant main effect of the set size (*F*(1, 3) = 15.9, *P*<0.05), with no effect of the waveform (*F*(1, 3) = 2.26, *P* = 0.207) and no significant interaction (set size x waveform) effect (*F*(3, 1) = 0.133, *P* = 0.733). The set-size effect is plotted in Figure 3b. The small difference between the sine- and square-wave conditions is not significant.

#### Preliminary discussion

In the Experiment 2, we found no significant difference between the two modulation conditions. Both sine- and square-wave conditions led to significant and comparable set-size effects. This confirms that the absence of a set-size effect in the square modulation condition of Experiment 1 can be attributed to the synchronized presence of a transient auditory signal. In addition, participants responded more quickly on the visual search task in Experiment 2 than in Experiment 1. This effect could be explained by participants using distinct conscious strategies for audiovisual and visual-only trials, as suggested in the Discussion of Experiment 1. If so, the facilitation in visual search observed in the square-wave condition of Experiment 1 could be due to a voluntary binding of visual and auditory information. To test this assumption, we used a cost–benefit paradigm in Experiment 3.

### Experiment 3

**Figure 5:**
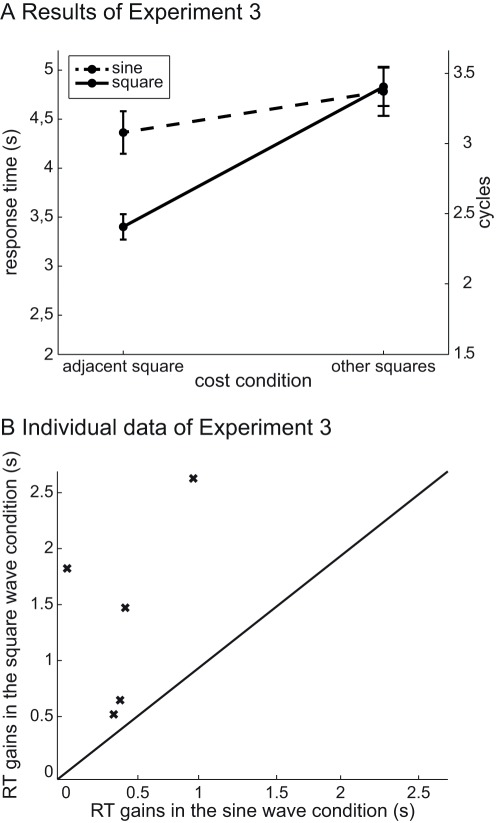
Results of Experiment 3. (A) Mean response times pooled across five participants as a function of cue validity and waveform. See legend from Figure 3 for details. (B) Individual results of Experiment 3. Response time gains (RT(other squares) - RT(adjacent square)) in the square-wave condition as a function of the response time gains in the sine-wave condition. See legend from Figure 4 for details.

As in the first two experiments, overall mean error rate was approximately 5% and error trials were discarded. A cut-off was applied at 2 standard-deviations from the mean response time for each participant (see Figure 5a and 5b and Table S3). A repeated-measures ANOVA was run on the response times with cue validity (valid vs. invalid) and waveform (sine-wave vs. square-wave) as within-subject variables. The ANOVA revealed a significant effect of cue validity (*F*(1, 3) = 15.3, *P*<0.05), no effect of the waveform (*F*(1, 3) = 2.84, *P* = 0.167) and a significant interaction (cue validity * waveform) effect (*F*(3, 1) = 8.47, *P*<0.05).

#### Preliminary discussion

The results of Experiment 3 (Figure 5a) show a clear benefit in the square- compared to the sine-wave condition when the sound was synchronized with the adjacent square, and a cost when the square-wave sound was synchronized with one of the other squares. Even though the sound correlated with the adjacent square in only 20% of the trials, which all observers knew, results suggest that observers were unable to stop using the audiovisual synchrony. In 80% of trials, this strategy led to a wrong square and consequently slowed down the visual search process. This cost effect implies that the audio-visual correlation was automatically bound and could not be easily ignored.

## Discussion

The goal of this series of experiments was to explore the effect of an auditory cue on visual search for stereomotion-defined visual stimuli. In the first two experiments, we showed that an amplitude-modulating auditory beep synchronized with a visual target led to efficient visual search. On the face of it, this result seems to contradict the finding from Harris, et al. [5] that stereomotion does not pop out. Moreover, we found a significant improvement in visual search only when the auditory and visual modulations were square and not sine. Our results add to those obtained by van der Burg et al. [10] by showing that pip and pop is neither the exclusive domain of the luminance system, nor is it purely monocularly-driven.

Our predictions were that, contrary to the luminance system, the stereo system would be more efficient at tracking smooth (sine-wave) rather than abrupt (square-wave) changes of disparity over time. Instead, we found that visual search was more efficient for square-wave than for sine-wave modulations of depth. This suggests that the stereo system is better able to keep track of rapid temporal modulations in spatio-temporal disparity when guided by an auditory cue.

The third experiment was aimed at investigating whether the results from Experiments 1 and 2 could be attributed to an automatic integration of auditory and visual temporal signals or to a voluntary attention-like effect. The results of this last experiment suggest that even when the sound led to wrong locations and thus impaired visual search, the correlation between the auditory and visual signals could not be easily ignored. This conclusion is consistent with an interpretation in terms of audiovisual integration rather than one of crossmodal attention.

Neural structures differentially responsive to synchronized audiovisual events have been found throughout the human cortex [7]. Recently, luminance-driven pip and pop-related increases in event related potentials were observed over lateral occipital areas of cortex [11]. It is conceivable that the compulsory audio-visual integration we observe may be related to audio-visually evoked activity in similar cortical areas.

The results of the experiments described in this article suggest that three main conclusions. First, an auditory cue can significantly improve the detection of targets defined exclusively by stereomotion, and second, that the stereo system is able to track abrupt changes of disparity over time when it is paired with a synchronized auditory signal. Third, and more generally, our findings support the idea that the pip and pop effect is likely to be mediated at a cortical level as we have demonstrated it here with stimuli that are exclusively binocularly defined.

## Supporting Information

Table S1 **Individual data of Experiment 1.** Individual response times (s) as a function of set size and waveform for Experiment 1.
(DOCX)

Table S2 **Individual data of Experiment 2.** Individual response times (s) as a function of set size and waveform for Experiment 2.
(DOCX)

Table S3 **Individual data of Experiment 3.** Individual response times (s) as a function of cue validity and waveform for Experiment 3.
(DOCX)
