# Supplementary material for: Synchronized Audio-Visual Transients Drive Efficient Visual Search for Motion-in-Depth
Source: PLoS One. 2012 May 17;7(5):e37190. doi: 10.1371/journal.pone.0037190 (PMC3355117; doi:10.1371/journal.pone.0037190)
Supplement: Table S1 — Individual data of Experiment 1. Individual response times (s) as a function of set size and waveform for Experiment 1. (DOCX) [file pone.0037190.s001.docx]

**Table 1: Individual data of Experiment 1.**

|  | sine-wave | | square-wave | |
| --- | --- | --- | --- | --- |
|  | set size = 6 | set size = 10 | set size = 6 | set size = 10 |
| E.O.M. | 3.15 | 3.84 | 2.15 | 2.70 |
| T.A. | 2.74 | 3.22 | 2.17 | 2.39 |
| D.A. | 3.86 | 4.52 | 2.71 | 2.91 |
| J.C. | 2.87 | 4.01 | 2.65 | 2.84 |
| M.Z. | 2.03 | 2.51 | 2.15 | 2.05 |

Individual response times (s) as a function of set size and waveform for Experiment 1.
