# Supplementary material for: Synchronized Audio-Visual Transients Drive Efficient Visual Search for Motion-in-Depth
Source: PLoS One. 2012 May 17;7(5):e37190. doi: 10.1371/journal.pone.0037190 (PMC3355117; doi:10.1371/journal.pone.0037190)
Supplement: Table S2 — Individual data of Experiment 2. Individual response times (s) as a function of set size and waveform for Experiment 2. (DOCX) [file pone.0037190.s002.docx]

**Table 2: Individual data of Experiment 2.**

|  | sine-wave | | square-wave | |
| --- | --- | --- | --- | --- |
|  | set size = 6 | set size = 10 | set size = 6 | set size = 10 |
| E.O.M. | 2.32 | 2.61 | 1.95 | 2.23 |
| T.A. | 2.79 | 4.19 | 3.03 | 3.29 |
| D.A. | 2.45 | 3.49 | 2.08 | 3.34 |
| J.C. | 2.21 | 3.31 | 2.40 | 3.73 |
| M.Z. | 2.83 | 3.08 | 2.45 | 2.91 |
