# Supplementary material for: Synchronized Audio-Visual Transients Drive Efficient Visual Search for Motion-in-Depth
Source: PLoS One. 2012 May 17;7(5):e37190. doi: 10.1371/journal.pone.0037190 (PMC3355117; doi:10.1371/journal.pone.0037190)
Supplement: Table S3 — Individual data of Experiment 3. Individual response times (s) as a function of cue validity and waveform for Experiment 3. (DOCX) [file pone.0037190.s003.docx]

**Table 3: Individual data of Experiment 3.**

|  | sine-wave | | square-wave | |
| --- | --- | --- | --- | --- |
|  | adjacent square | other squares | adjacent square | other squares |
| E.O.M. | 3.47 | 3.90 | 2.50 | 3.98 |
| T.A. | 5.15 | 5.50 | 4.06 | 4.59 |
| D.A. | 5.41 | 6.31 | 3.58 | 6.22 |
| J.C. | 3.25 | 3.64 | 3.61 | 4.27 |
| M.Z. | 4.54 | 4.56 | 3.26 | 5.09 |
